# Urosepsis From Nephrolithiasis Caused by Candida glabrata: A Rare Etiology of Urinary Sepsis in an Immunocompetent Patient

**DOI:** 10.7759/cureus.84600

**Published:** 2025-05-22

**Authors:** Jonathan Van Name, Krunal Shukla

**Affiliations:** 1 Internal Medicine, College of Medicine, University of Florida, Gainesville, USA

**Keywords:** candida, infectious disease, mycology, nephrolithiasis, urosepsis

## Abstract

Nephrolithiasis can often serve as an infection nidus, as it is typically associated with urease-producing bacteria and can obstruct the urinary tract, leading to pyelonephritis. While most urinary tract infections from septic stones are bacterial in etiology, fungal causes are less commonly reported in immunocompetent patients and rarely manifest as fungemia. Among fungal causes, *Candida albicans *is the most common. We describe the case of a 41-year-old immunocompetent patient with a history of recurrent kidney stones and percutaneous nephrostomy tube placement, who developed sepsis from a urinary source secondary to a nephrolithiasis. Blood culture isolates grew *Candida glabrata*. Following identification of the fungus, the patient received a two-week intravenous micafungin course and outpatient stone removal per urological surgery.

## Introduction

Sepsis from nephrolithiasis is a serious medical condition that predisposes patients to a high risk of uroseptic shock and acute kidney injury [1]. While the most common causes of urinary tract infection (UTI) from kidney stones are bacterial in etiology (e.g., Escherichia coli, Proteus, and Klebsiella), fungi have increasingly been reported as etiologic agents in kidney stone-induced sepsis [2]. The increasing frequency of diabetes and poor glycemic control is likely a contributing factor in the prevalence of fungal UTIs. Although *Candida albicans* is the most common cause of a fungal UTI, *Candida glabrata* has rarely been reported to cause UTI-related sepsis in immunocompetent patients [3].

*Candida glabrata *is an asexual, haploid yeast that is commonly found in the environment, particularly on flowers, water, and soil. It typically infects and colonizes epithelial surfaces, most commonly in the mouth, gastrointestinal tract, and vaginal surfaces [4]. *Candida glabrata* is the second most isolated Candida species, possessing important medical relevance in critically ill patients, owing largely to the increasing prevalence of acquired immunodeficiency syndrome (AIDS), malignancy, and uncontrolled diabetes mellitus [4]. *Candida glabrata* has been implicated in 15%-25% of invasive fungal clinical cases but rarely manifests as the causative pathogen for urinary sepsis [5].

Although bacterial etiologies are more common pathogens than fungal causes in UTIs, the underlying pathophysiology of nephrolithiasis explains their existence. Due to obstruction (full or partial) of urinary structures, urinary stasis itself can serve as an infection nidus and can create an environment conducive to the growth of fungal species even in immunocompetent patients. The present report describes the case of a man with fungemia from Candida glabrata caused by a nephrolithiasis-induced UTI.

## Case presentation

**Figure 1:**
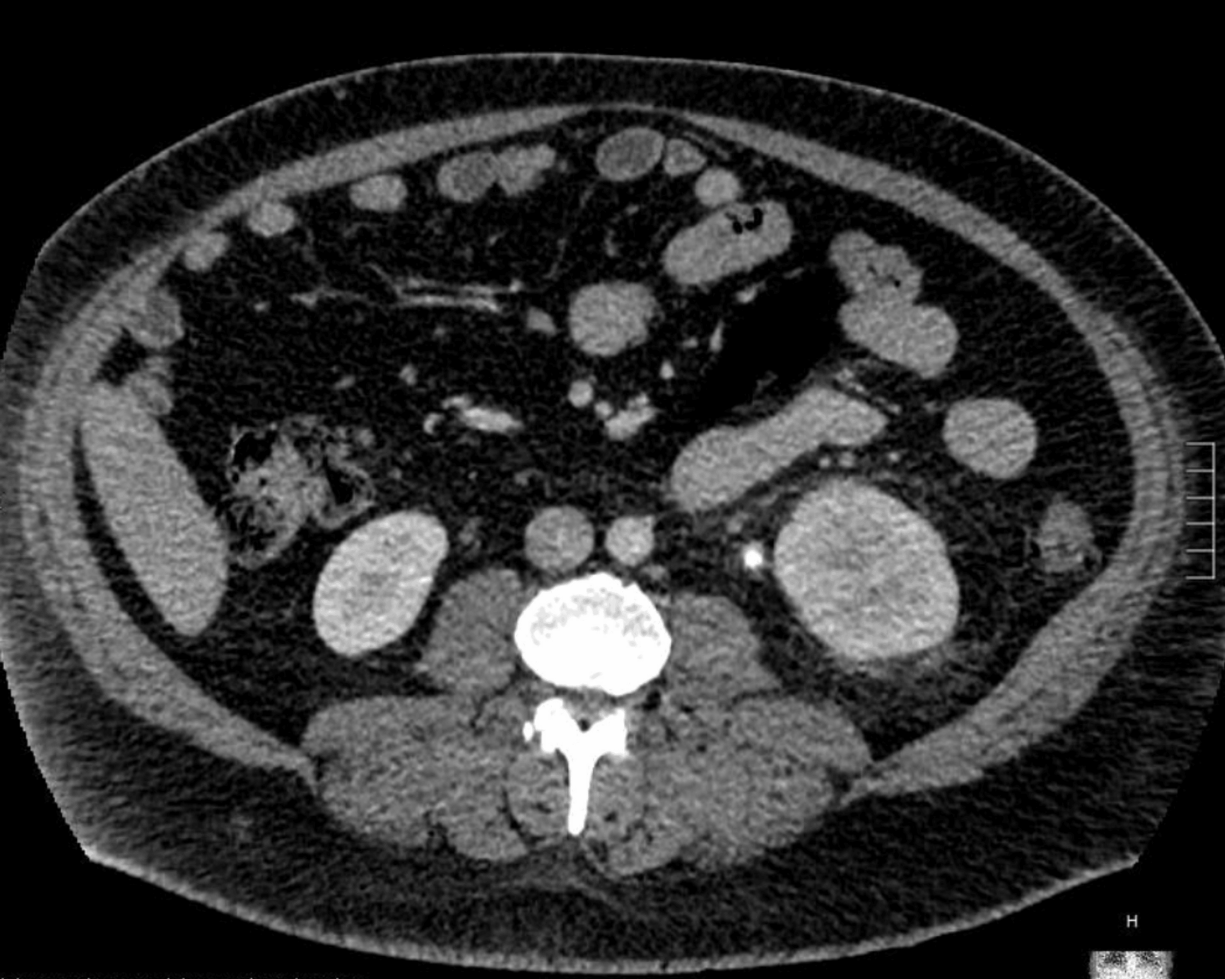
CT abdomen/pelvis showing proximal left ureteral stone, moderate left-sided hydronephrosis, and left perirenal stranding.

**Figure 2:**
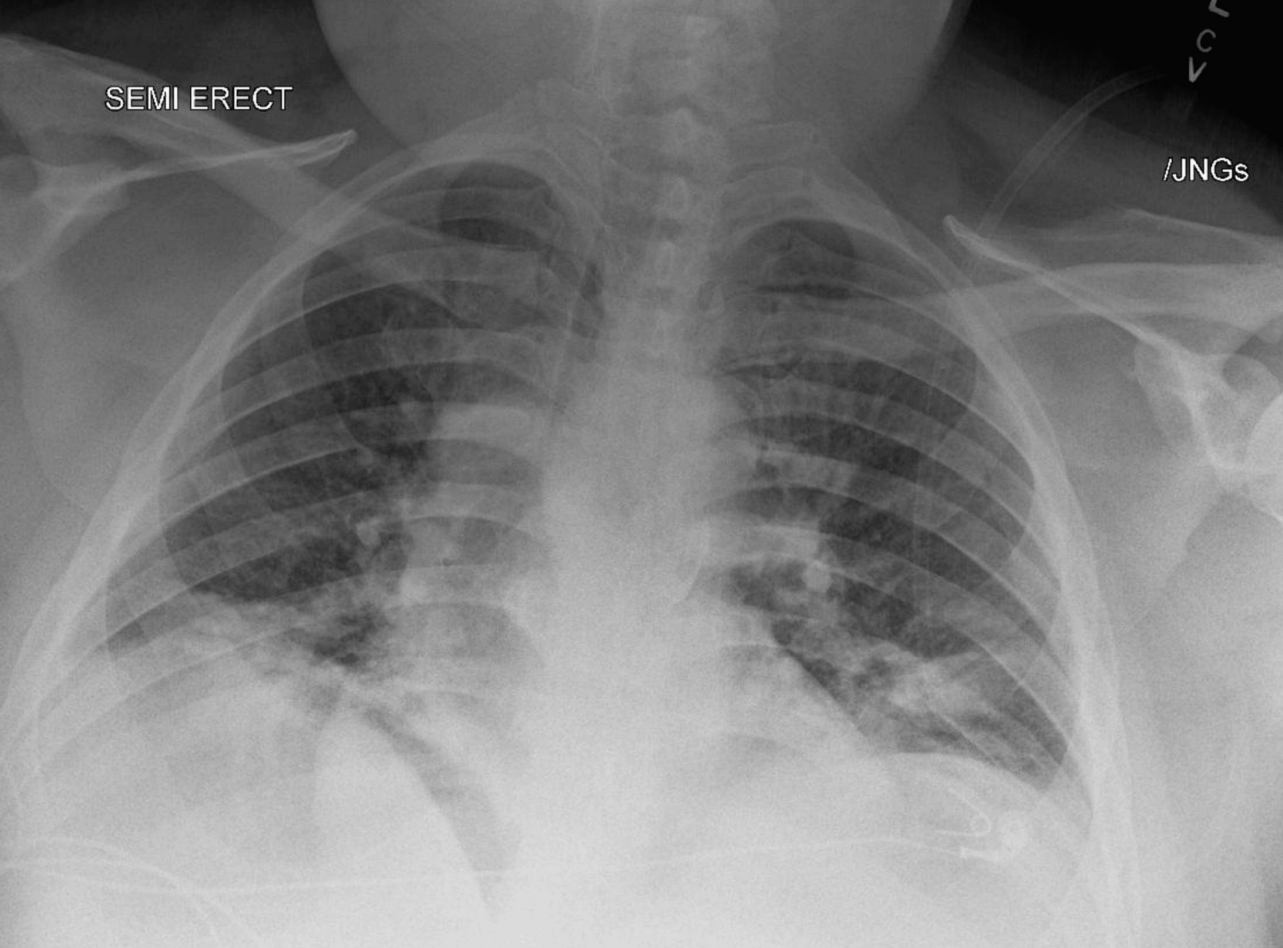
Chest X-ray demonstrating bibasilar atelectasis and mild fluid overload causing pulmonary edema.

A 41-year-old male with a history of well-controlled diabetes mellitus, hypertension, and recurrent kidney stones (the most recent occurring three months before admission) presented to the hospital with subjective fever and bilateral flank pain in the setting of a recent kidney stone. In the Emergency Department, the patient was normotensive, tachycardic at 116 beats per minute, with a respiratory rate of 19 breaths per minute, oxygen saturation of 96% on room air, and a fever of 103.2 °F. Additionally, his complete blood count (CBC) demonstrated a leukocytosis, with a white blood cell count of 17.0 x 10^3^/uL and a neutrophil count of 14.09 x 10^3^/uL. The patient had a CT abdomen/pelvis image that showed a 3.5 x 0.6 cm obstructing stone in the proximal left ureter, causing moderate left-sided hydronephrosis, as well as left perirenal stranding (Figure 1). A urinalysis collected on admission demonstrated moderate leukocyte esterases, 119 WBC/HF, a small amount of blood, and negative nitrates, with a reflexive urine culture showing no growth of any organisms. Additionally, a chest X-ray showed mild fluid overload with bibasilar atelectasis (Figure 2). Given imaging evidence of an obstructing stone in the setting of positive systemic inflammatory response syndrome (SIRS) criteria, the patient was diagnosed with UTI sepsis and started on broad-spectrum antibiotics with ceftriaxone. Additionally, the patient was found to have an acute kidney injury with creatinine of 4.1 (baseline of 1.0-1.1), which was attributed to obstructive pathology.

As the patient’s hospital course progressed to day 3, the patient continued to be febrile even after 48 hours of ceftriaxone therapy. Blood cultures were obtained, and the patient’s antibiotics were broadened to include vancomycin and piperacillin/tazobactam (Zosyn) given ceftriaxone failure. Additionally, the urological surgery and interventional radiology (IR) services were consulted for hydronephrosis secondary to nephrolithiasis, and IR placed a left-sided percutaneous nephrostomy tube, resulting in resolution of the obstructive acute kidney injury. On day 3 of the hospital course, the patient’s blood cultures were positive for *Candida glabrata *(with no available sensitivity data), and the Infectious Disease service was consulted for antifungal regimen recommendations. Notably, the patient's urine cultures showed no growth. After clearance of blood cultures on hospital course day 5, a peripherally inserted central catheter (PICC) was placed, and the patient was discharged with a two-week course of intravenous (IV) micafungin therapy, as sensitivity data were not obtained. 

The patient followed up closely with urology and IR in the outpatient setting. Given the patient’s clinical improvement on IV micafungin, the urology team deferred surgical intervention for the left-sided nephrolithiasis. Per the IR service, the patient continued to have the percutaneous nephrostomy tube in place for a minimum of three months for kidney decompression. Additionally, the patient was instructed to follow up closely with his primary care provider for strict glycemic control in the context of his diabetes and fungal infection.

## Discussion

*Candida glabrata* fungemia, though rare, is an increasingly recognized pathogen in critically ill and septic patients who fail to improve after initial antibiotic therapy. While previous case reports exist demonstrating candidal nephrolithiasis and subsequent systemic infections, they focus on *Candida albicans* as the source of the fungemia.This case report demonstrates the importance of suspecting fungal pathogens in non-resolving nephrolithiasis-induced infections and further highlights the role of *Candida glabrata* in these infections. Based on the literature review, it is critically important to recognize fungal findings on both urine and blood cultures, as previous case reports demonstrate that positive fungal culture findings are often misattributed as being contaminants*. *

The risk factors for *Candida glabrata* infection include the use of indwelling catheters, recent antibiotic use, uncontrolled glycemic control, and an immunocompromised/debilitated host [6]. While the patient presented in this case was immunocompetent, the patient’s history of percutaneous nephrostomy tube placement and previously uncontrolled diabetes placed him at an increased risk of *Candida glabrata* fungemia. Thus, this case further illustrates the risk factors associated with fungal bloodstream infections, including urinary stasis secondary to obstruction from a kidney stone.

Although the patient in this case report recovered well with IV micafungin and percutaneous nephrostomy tube placement, there is growing concern amongst the Infectious Disease community about an increasingly high prevalence of *Candida glabrata* antifungal resistance. Additionally, previous case reports have highlighted this concern in UTIs, specifically regarding echinocandin resistance in *Candida glabrata *strains with *FKS *mutants. However, the current Infectious Disease Society of America (IDSA) guidelines recommend initially treating *Candida glabrata* like other Candida species fungemia. The treatment approach is highly dependent on the neutrophil count of the patient. For non-neutropenic patients, the IDSA recommends either a two-week course of echinocandin (e.g., micafungin) therapy in moderately severe to severe cases, or fluconazole in less severe infections [7]. Neutropenia warrants more intensive therapy with either liposomal amphotericin B or an echinocandin for a minimum of two weeks [7]. However, given increasing echinocandin resistance to *Candida glabrata* compared to *Candida albicans*, the management of *Candida glabrata* is often more challenging [8].

In this case report, it is uncertain whether the *Candida glabrata *infection originated from colonization of the percutaneous nephrostomy tube or was secondary to urinary stasis from nephrolithiasis obstruction. However, it remains possible that both tube colonization and urinary tract involvement were present in this case to induce systemic infection in this immunocompetent patient. This case report demonstrates the importance of early administration of antifungal therapy, surgical evaluation for source control (i.e., percutaneous nephrostomy tube placement with an outpatient plan for stone removal), and recommendations from the Infectious Disease consultants. 

## Conclusions

Urosepsis due to systemic *Candida glabrata *infection in an immunocompetent individual is incredibly rare. While there are a few reports in medical literature that demonstrate *Candida glabrata *sepsis from urinary source, most of these cases are in patients with either recent abdominal/pelvic surgeries or in immunocompetent patients undergoing cancer treatment. This case report underscores the importance of early recognition and aggressive management of *Candida glabrata* fungemia in patients with UTIs secondary to obstructive nephrolithiasis. Based on this report, clinicians should have a high index of suspicion for fungal infection in patients with urosepsis who fail to improve with antibiotic therapy. Additionally, clinicians should have a low threshold for empiric antifungal coverage in patients with a known fungemia history or patients with significant risk factors for opportunistic infection. Prompt source control remains paramount in pathogen eradication, as fungal infections are unlikely to resolve without addressing the underlying nidus. Given the aggressive resistance profile of *Candida glabrata*, further studies are needed to evaluate optimal antifungal therapy duration and treatment strategies for patients with *Candida glabrata* fungemia.
